# Recent Advances in Dielectric Elastomer Actuator-Based Soft Robots: Classification, Applications, and Future Perspectives

**DOI:** 10.3390/gels11110844

**Published:** 2025-10-22

**Authors:** Shuo Li, Zhizheng Gao, Wenguang Yang, Ruiqian Wang, Lei Zhang

**Affiliations:** School of Electromechanical and Automotive Engineering, Yantai University, Yantai 264005, China; 13287341873@163.com (S.L.); gaozhizheng2024@163.com (Z.G.); yangwenguang@ytu.edu.cn (W.Y.); wrq0969@163.com (R.W.)

**Keywords:** dielectric elastomer actuators (DEAs), soft robots, soft elastomers, intelligent sensing

## Abstract

With the growing application of soft robot technology in complex, dynamic environments, the limitations of traditional rigid robots have become increasingly prominent, urgently demanding novel soft actuation technologies. Dielectric elastomer actuators (DEAs) have gradually emerged as a research focus in soft robotics due to their high energy density, rapid response, low noise, and excellent compliance. This paper systematically reviews the research progress of DEA-based soft robots over the past decade. Using classification and comparative analysis, DEAs are categorized into four basic types according to their initial shape—planar, saddle-shaped, cylindrical, and conical—with detailed elaboration on their working principles, structural features, and typical applications. Furthermore, from two major application scenarios (underwater and terrestrial), this paper analyzes the adaptability of various DEAs in robot design and corresponding optimization strategies and summarizes their performance and research challenges in bionic propulsion, multi-modal motion, and environmental adaptability. Finally, it provides the prospective future research directions of DEAs in material development, structural design, intelligent control, and system integration, providing theoretical support and technical references for their wide application in fields such as medical treatment, detection, and human–robot interaction.

## 1. Introduction

Since the mid-20th century, robotic technology has advanced from concept to reality, with “rigidity” dominating its research for a long time. Traditional rigid robots, composed of metal structures, gears, bearings, and traditional motors, rely on precise rigid joints for movement [1,2,3,4,5], but their inherent rigidity brings limitations: poor adaptability to dynamic/complex environments, potential human–robot interaction safety hazards, and difficulty simulating biological, compliant movements [6,7]. These constraints restrict their use in medical rehabilitation [8], disaster relief and detection [9], and human–robot collaboration [10]. Inspired by the deformability and environmental adaptability of soft-bodied organisms (e.g., octopuses, jellyfish), soft robots have emerged as a new research direction [11,12]. Soft robots are mainly constructed from highly compliant materials, including shape memory alloys (SMAs) [13], hydrogels [14], dielectric elastomer actuators (DEAs) [15], and ionic polymer–metal composites actuators (IPMC) [16]. Through bionic structural design and the adoption of driving mechanisms such as fluid-driven [17], smart material-driven [18], chemical reaction-driven [19], and pneumatic-driven [20], they achieve continuous, jointless, large-scale deformable movement. Their softness endows them with high environmental adaptability (for interacting with fragile objects like biological tissues), good human–robot collaboration safety, impact resistance [21], and self-recovery capabilities [22], expanding robotics applications and driving its evolution toward intelligence, bionics, and integration.

The core challenge in soft robotics lies in developing soft actuators that simultaneously exhibit large deformation [23], high output force [24], fast response [25], and good encapsulation. Currently, a variety of soft actuation technologies have been extensively explored. Luo et al. proposed a jellyfish-like robot based on SMA materials [26]. This robot overcomes the actuation limitations of traditional SMA materials by using SMA springs as actuators, fully leveraging the large deformation advantage of SMAs to enhance contraction and recovery performance. Compared with robots driven by filamentous or sheet-like SMA materials, this actuator can more accurately simulate the bell-shaped oscillation of jellyfish [27,28]. Experimental results show that the robot can generate a maximum force of 1.62 N and achieve a maximum underwater movement speed of 0.33 body lengths per s (BL/s). Additionally, multi-modal movements (e.g., linear motion and steering) can be realized by controlling the energization sequence of different actuators. However, SMA performance is highly sensitive to ambient temperature, and the thermal cycle efficiency of the actuator is low. Particularly during the cooling phase for shape recovery, it relies solely on water cooling—limiting its maximum actuation frequency. Wang et al. proposed a pneumatically driven jellyfish-like robot [29]. This robot uses a pneumatic bistable actuator (PBA) as its core driving unit, marking the first application of a pneumatic bistable structure in underwater bionic robots. Owing to its bistable characteristics, an external energy supply is only required during state switching, with no continuous energy consumption. Experimental results demonstrate that the PBA pneumatic actuation structure [30,31] is simple and provides a stable driving force: the robot achieves a maximum underwater swimming speed of 0.38 BL/s and can operate stably even with a 1.19 kg load. However, pneumatic actuation is affected by external pressure in high-pressure deep-water environments; the TPU film it relies on is prone to tensile deformation after repeated high-pressure inflations, reducing actuation stability. Furthermore, pneumatic actuation makes it difficult to control the robot’s attitude for multi-modal movements such as steering and pitching. Yin et al. proposed a jellyfish-like robot based on hydrogel [32]. This study introduces visible light driving into the research of jellyfish-like soft robots for the first time. By incorporating carbon nanotube CNTs [33] into PNIPAM hydrogel, the photothermal conversion efficiency and response speed of the material are significantly improved, enabling rapid and reversible deformation under visible light irradiation. However, the swimming speed of the robot is relatively low, with a maximum underwater swimming speed of only 3.37 mm/s.

In contrast, DEAs have gained prominence due to their high energy density, millisecond-level response, low noise [34], and high compliance (Table 1), which have been applied in lots of soft robots. For example, Du et al. proposed a thin vibrating crawling robot driven by a planar DEA [35]. The overall structure of the robot is composed of flexible DEA actuation and soft bristles, without rigid transmission components. It has extremely low operating noise and a maximum resonant frequency of 156 Hz during operation, with a relatively fast response speed. Goh et al. focused on studying the super-large electroinduced strain performance of acrylic DEA. Through theoretical analysis and experimental verification, they broke through the deformation limitations of traditional soft actuators, and the maximum actuating strain can reach 500% [36]. The basic structure of a DEA can be abstracted as a “sandwich”-type composite structure [37]. Its core is an ultra-thin, highly elastic insulating material (e.g., acrylic or silicone rubber), and flexible conductive electrodes coat the elastomer’s upper/lower surfaces; actuation relies on the Maxwell stress effect [38,39], and a high-voltage electric field (kV level) generates electrostatic attraction, squeezing the dielectric layer to thin it (thickness direction) and expand its planar area (generating strain/force).

**Figure 1:**
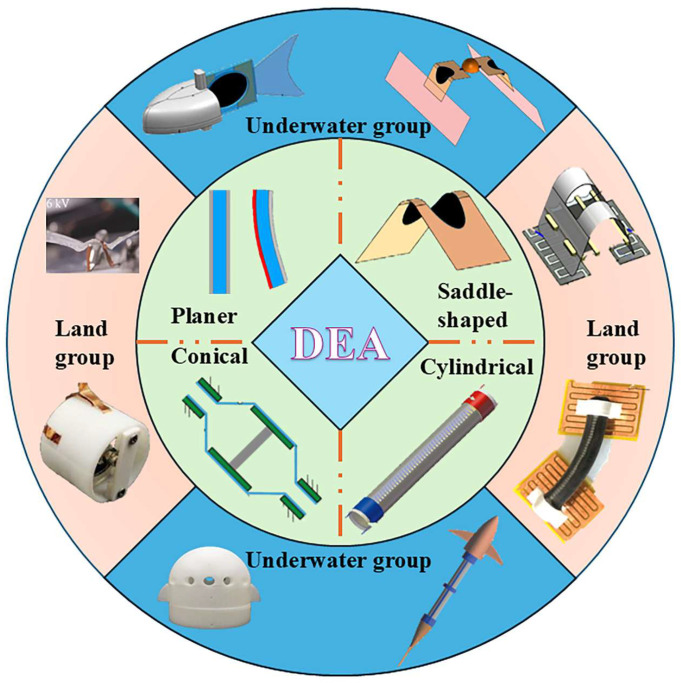
Article content roadmap. Planar: Reproduced from Reference [55] with permission from the *Journal of Physics: Conference Series*. Saddle-shaped: Reproduced from Reference [56] with permission from *Ocean Engineering*. Cylindrical: Reproduced from Reference [57] with permission from *Soft Robotics*. Conical: Reproduced from Reference [58] with permission from *Micromachines*. Land group: Reproduced from Reference [58] with permission from *Micromachines*. Reproduced from Reference [59] with permission from *NPG Asia Materials*. Reproduced from Reference [60] with permission from *Extreme Mechanics Letters*. Reproduced from Reference [61] with permission from *Advanced Intelligent Systems*. Underwater group: Reproduced from Reference [55] with permission from the *Journal of Physics: Conference Series*. Reproduced from Reference [56] with permission from *Ocean Engineering*. Reproduced from Reference [57] with permission from *Soft Robotics*. Reproduced from Reference [62] with permission from *Advanced Engineering Materials*.

Despite these advantages, the performance of DEAs is profoundly influenced by their initial geometric configuration. Different shapes—such as planar, saddle-shaped, cylindrical, and conical—endow DEAs with distinct mechanical outputs and kinematic behaviors, making them suitable for specific robotic functions and environments. For example, saddle-shaped DEAs are well-suited for crawling robots, while cylindrical or conical designs can generate more complex, multi-degree-of-freedom motions ideal for underwater propulsion or grasping. This review emphasizes a structural taxonomy of DEAs based on their initial shapes, systematically examining how each configuration—planar, saddle-shaped, cylindrical, and conical—affects actuation performance and robotic application. The novelty of this approach lies in its structured comparison across geometries, which has not been comprehensively addressed in previous reviews. By categorizing DEA-driven robots according to both structure and operational environment, this manuscript aims to provide a clear design guideline for selecting DEA configurations tailored to specific robotic tasks. The structure of this paper is as follows: (1) Analysis of DEA performance factors, including voltage, frequency, and temperature. (2) Classification of DEAs by initial shape and review of corresponding robotic systems. (3) Discussion of DEA applications in terrestrial and underwater robots. (4) Future prospective DE development. The content roadmap is shown in Figure 1.

## 2. Factors Influencing the Performance of Dielectric Elastomer Films

The output characteristics of soft robots are influenced by multiple factors, primarily encompassing driving structure, applied external stimuli, and the intrinsic properties of the base material. For DEAs, deformations generated by different driving structures vary significantly; even with the same structure, external stimuli and base material properties still impact their deformation outcomes. The external stimulus for DEAs is an electrical signal, consisting of driving voltage and frequency. Additionally, pre-stretching (a common step in DEA fabrication) affects the properties of different matrix materials. Thus, actuation voltage, frequency, and pre-stretching are the core factors governing DEA motion performance.

### 2.1. Effect of Actuating Voltage on the Performance of DEAs

Per Maxwell’s stress calculation formula:

(1) $$\sigma =\varepsilon_{0}\varepsilon_{\gamma }{\left(\frac{U}{t}\right)}^{2}$$

where σ is the Maxwell stress, $\varepsilon_{0}$ is the vacuum dielectric constant, $\varepsilon_{\gamma }$ is the dielectric constant of the dielectric elastomer, U is the input voltage, and t is the thickness of the dielectric elastomer. Maxwell’s stress is directly proportional to the square of the applied voltage and inversely proportional to the square of the film thickness. Thus, a higher applied voltage leads to greater electrostatic pressure, which exerts a stronger squeezing effect on the elastomer film—thinning it in the thickness direction and causing significant expansion in the outer surface direction. Theoretically, the area strain is proportional to the square of the voltage [63].

However, applied voltage is not arbitrarily adjustable; it is strictly limited by the material’s dielectric breakdown strength [64,65]. Dielectric breakdown strength refers to the maximum electric field a material can withstand. Exceeding this limit causes instantaneous loss of insulation: high-voltage arcs destroy the molecular structure and form permanent conductive channels, leading to complete device failure. Under high voltage, uneven local electric fields (especially at material defects) significantly increase breakdown risk. Thus, practical working voltage is typically set well below the breakdown threshold [66]. Common DEA materials include acrylic films (e.g., VHB) and silicone rubber. Acrylic films exhibit high electrical strain (300%) but require pre-stretching; they have relatively low Young’s modulus and obvious viscoelasticity. Silicone rubber, by contrast, is easy to fabricate but has lower driving strain than acrylic films; it offers high dielectric strength and excellent reliability. These films generally require kV-level actuation voltage [67], but only microampere-level driving current. Most actuators are sealed, posing no significant human safety risks, with typical driving electric fields ranging from 1 MV/m to 100 MV/m [68,69,70]. In practice, voltage is kept below the maximum driving value to ensure long-term stable operation.

Different voltage sources (DC, AC, sinusoidal) affect dielectric elastomers differently. Sheng et al. investigated the viscoelastic responses of VHB films under DC, ramp, and sinusoidal voltages, finding that a lower ramp voltage rate corresponds to a higher critical stretch, and sinusoidal voltage induces greater deformation at low frequencies [71]. Kuhnel et al. compared the arc resistance of silicone under DC and AC stress, observing that DC stress causes higher temperatures and shorter endurance times, while electrode materials significantly influence arc mobility [72].

In summary, voltage acts as the “power source” for dielectric elastomers, directly determining output strain and force. Yet, its application is strictly constrained by the dielectric breakdown threshold. A core challenge in advancing DEA technology toward practical use is approaching this physical limit while ensuring stability and service life.

### 2.2. Effect of Actuating Frequency on the Performance of DEAs

Driving frequency is another key factor influencing the dynamic performance of dielectric elastomers. Per the Cole–Cole model [73], the dielectric constant of dielectric elastomers decreases significantly as frequency increases. From Formula (1), DEA output characteristics are directly proportional to dielectric constant—meaning higher frequency degrades output performance [74]. Dielectric elastomers are viscoelastic materials [75], and the movement/rearrangement of their molecular segments requires time. When a voltage signal is applied, deformation does not occur instantaneously; instead, it undergoes a delayed ascending process before reaching a steady state. This response is described by a time constant related to the material’s viscoelastic relaxation time. When the driving frequency is much lower than the reciprocal of the time constant, the material has sufficient time to respond fully, with deformation synchronizing to voltage changes and maximum amplitude similar to static driving. When frequency approaches the reciprocal of the time constant, molecular segments cannot fully rearrange, reducing strain amplitude and causing phase lag relative to the driving signal (i.e., deformation lags behind voltage changes). When frequency is much higher than the reciprocal of the time constant, the material barely responds to rapid electric field changes, resulting in minimal deformation and severely degraded output [76]. Microscopically, internal friction within the material causes energy loss. At low frequencies, molecular chain segment motion is relatively unimpeded, leading to low loss. As frequency increases, segment motion is restricted, friction intensifies, and loss rises significantly—even generating substantial heat. Accumulated heat accelerates material degradation and shortens service life. Thus, selecting an appropriate driving frequency is critical to maximizing dielectric elastomer performance.

### 2.3. Effect of Pre-Stretching on the Performance of DEAs

Pre-stretching—defined as applying mechanical tension to a dielectric elastomer film prior to voltage application, fixing it in a stretched state, and allowing it to fully relax—is one of the most effective and widely adopted methods for optimizing electro-driven performance.

Electrical breakdown is the primary failure mode of dielectric elastomers, and pre-stretching significantly increases this threshold [77,78]. Common dielectric films (e.g., VHB) inevitably contain micro-defects during fabrication (e.g., micro-bubbles, impurities, micro-cracks, uneven thickness), which cause local electric field concentration and act as breakdown initiation points. Pre-stretching acts as a “screening process”: most weak defects either fracture in advance or undergo irreversible deformation, being “eliminated” or “passivated”. Additionally, pre-stretching uniformizes film thickness, reducing electric field unevenness caused by thickness fluctuations.

Pre-stretching determines the film’s initial mechanical state, thereby influencing actuation behavior [79]. Uniaxial pre-stretching (stretching in one direction) induces anisotropic mechanical properties—a higher modulus in the stretching direction than in the vertical direction. Under voltage, area expansion also becomes anisotropic: strain perpendicular to the pre-stretching direction is much larger than that parallel to it. In contrast, biaxial pre-stretching (stretching in two mutually perpendicular directions) maintains in-plane isotropy, enabling more uniform planar expansion. Pre-stretching affects different materials differently. For example, acrylates are highly sensitive to pre-stretching: Pelrine et al. were the first to report that VHB4910 achieved 158% area expansion under 300% pre-stretching, laying the foundation for pre-stretching technology [46]. For silicone rubber, however, stretching induces siloxane chain orientation; excessively high pre-stretching ratios reduce dielectric constant and thus output performance. Pre-stretching is a trade-off: it sacrifices partial mechanical simplicity and introduces time-dependent behavior, but in return, it significantly improves electrical and actuation performance.

Finally, the failure of dielectric elastomers is also an important research aspect. Its failure can mainly be attributed to three aspects: electrical breakdown, mechanical fatigue, and electromechanical instability. To enhance stability and service life, multi-dimensional collaborative optimization is essential: at the material level, self-healing elastomers can be developed, molecular hybrid networks can be designed, or functional composite materials can be introduced to improve dielectric properties and increase dielectric constants, avoiding charge accumulation and electrical breakdown, thereby reducing the driving voltage while enhancing the material’s inherent toughness. At the drive control level, the pre-stretching strategy and drive waveform should be optimized to suppress viscoelastic relaxation and alleviate the phenomenon of strain localization. At the structural design level, it is necessary to rationally design the actuator configuration and the shape of the flexible electrode to avoid stress concentration and ensure the consistency and reliability of the electromechanical response. Through systematic optimization in the three aspects of materials, drive, and structure, the comprehensive performance and long-term service stability of the actuator can be significantly improved. In conclusion, understanding these influencing factors provides critical guidance for soft robot design. After selecting a base material, adjust the pre-stretching ratio based on functional requirements (different materials require different ratios). Determine voltage amplitude and frequency according to experimental conditions and required maximum output performance, then tune these parameters within safe ranges to meet experimental performance targets.

## 3. Planar DEAs

### 3.1. Working Principle of Planar DEAs

**Figure 2:**
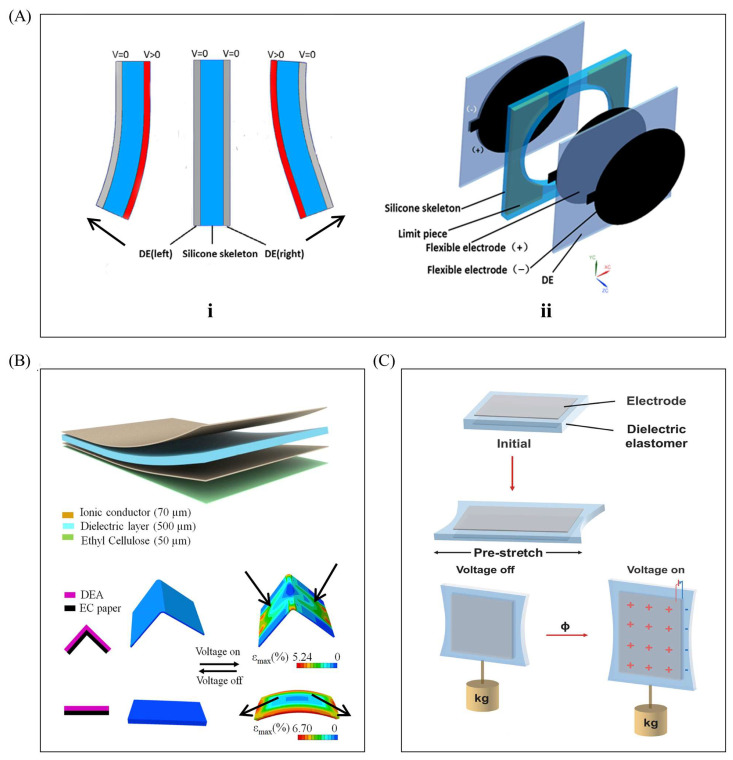
Planar DEAs. (**A**) Double-layer planar DEA. Reproduced from Reference [55] with permission from the *Journal of Physics: Conference Series*. (**B**) Driving structure combining origami material and DE material. Reproduced from Reference [59] with permission from *NPG Asia Materials*. (**C**) A planar actuator made of a new composite dielectric elastomer material. Reproduced from Reference [69] with permission from *Nature Communications*.

The actuation component of a planar DEA has a relatively simple structure. Its basic unit typically consists of a stretched dielectric film, a rigid layer, and two conductive layers. When voltage is applied, charges accumulate in the two conductive layers (sandwiching the dielectric layer), causing the dielectric layer to deform. However, the rigid layer restricts this deformation, leading to bending of the DEA. Key planar DEA designs (Figure 2) are as follows: Dual-layer planar DEA (Figure 2(Ai)): Pre-stretched dielectric layers attach to both sides of a rigid layer. Energizing one dielectric layer induces expansion, while the rigid frame and non-energized layer remain dimensionally unchanged—creating a mismatch that causes bending. Sequential voltage excitation of both layers enables continuous oscillation. Rigid silicone-supported DEA (Figure 2(Aii)): Two dielectric layers (with flexible electrodes on both sides) sandwich a rigid silicone layer (for support). A cutout (matching the electrode area) is made in the silicone layer to ensure axial bending. Origami-integrated DEA (Figure 2B): Comprises one dielectric layer (the actuation part, with ionic conductors as flexible electrodes for uniform energization) and one rigid origami layer (driven part). Voltage-induced expansion of the dielectric layer bends the actuator toward the origami layer (which is voltage-insensitive); a middle crease in the origami layer (low stiffness after bending) enhances bending on both sides of the crease. Composite dielectric DEA (Figure 2C): Uses a new composite dielectric film with a modified molecular structure for enhanced mechanical properties. Its characteristics are validated via planar DEA actuation tests.

### 3.2. Robots Based on Planar DEAs

**Figure 3:**
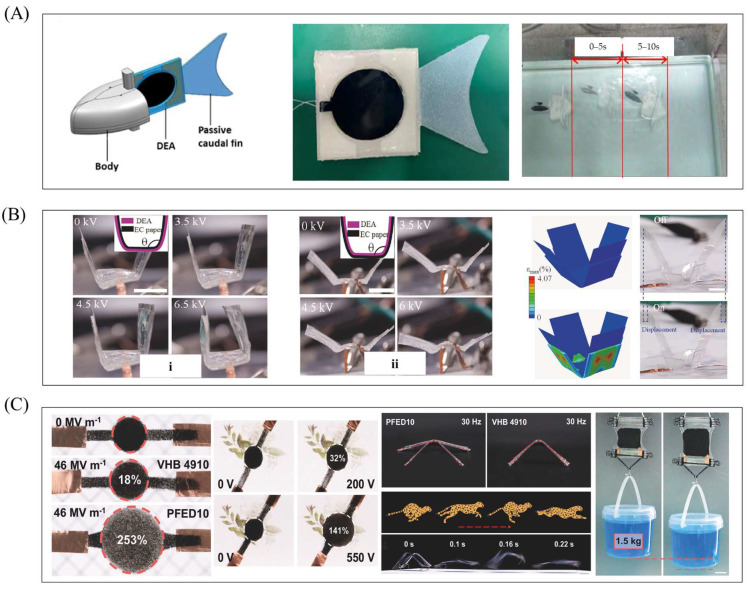
Robots based on planar DEAs. (**A**) The structure and movement process of the BCF propulsion mode soft robotic fish. Reproduced from Reference [55] with permission from the *Journal of Physics: Conference Series*. (**B**) Two actuator structures combining DE film and origami materials, and the simulation results. Reproduced from Reference [59] with permission from *NPG Asia Materials*. (**C**) A performance comparison between a dielectric elastomer of a composite material with large strain and ultra-high energy density and the VHB4910 material. Reproduced from Reference [69] with permission from *Nature Communications*.

This section will focus on typical robot cases based on planar DEA, systematically introducing its specific research achievements in bionic motion, scenario-based functional implementation, and innovative material applications and demonstrating the application potential of planar DEA in low-load and high-frequency response scenarios. Planar DEAs generate less deformation and output force and are typically suitable for the design of underwater robots and land-based micro-robots. For example, the body and caudal fin (BCF) propulsion mode is the fastest swimming method for fish. The actuation part, composed of dielectric elastomers (a type of “artificial muscle” soft material), can simulate fish swimming well. Jiao et al. proposed a BCF bionic robotic fish (Figure 3A) driven by dielectric elastomers, based on the actuator in Figure 2A [55]. The robotic fish has an inverted triangular overall shape: its head and passive caudal fin are fabricated via 3D printing, while the middle actuation part adopts a dielectric elastomer actuation structure. This robotic fish mimics the BCF movement mode of real fish [80]: during swimming, the front part of the body has a small oscillation amplitude, and the movement of the passive caudal fin is mainly driven by the oscillation of the rear body part. The actuation part uses silicone resin as the skeleton (to simulate the fish spine) and dielectric elastomers (to simulate fish muscles). In experiments, a driving voltage of 3–7 kV was applied to the robotic fish. The results showed that the displacement of the DEA end oscillating to both sides was consistent, with a maximum displacement of 6.45 mm. The bending angle was positively correlated with voltage, and the sum of the bending angles on both sides reached a maximum of 31°. Only voltage affected the magnitude of the output force (driving frequency had no effect): as voltage increased from 3 kV to 7 kV, the output force increased from 7.13 mN to 48.54 mN. The maximum swimming speed in experiments reached 22.7 mm/s. This confirms that planar DEAs are highly suitable for designing robots adopting the BCF swimming mode.

Based on research on the basic structure of planar DEAs, many scholars have further expanded their application scenarios and developed actuation structures suitable for different fields. Among them, Wang et al. proposed two reconfigurable and programmable 3D deformation actuation structures, based on the actuator in Figure 2B [59]. Origami materials [81] typically complete movement via stiffness changes in local regions caused by origami patterns; when combined with dielectric elastomers (which deform under energization), the deformation of origami materials can be amplified. Using this combination method, two 3D deformation actuation structures were developed (Figure 3B), with their core difference lying in the combination mode: The actuation structure in Figure 3(Bi) adheres the dielectric elastomer layer to the outside of the origami material. The shape of the origami material dictates inward bending of the actuation structure; when energized, the outer dielectric elastomer layer expands in surface area, amplifying the inward bending deformation. The actuation structure in Figure 3(Bii) adheres the dielectric elastomer layer to the inside of the origami material. When energized, the dielectric elastomer layer expands in surface area, generating an outward bending effect. Although the origami material’s crease pattern tends to cause inward bending, the force from the dielectric elastomer layer can drive the origami material to bend outward. In this way, the entire structure exhibits a natural bending state when de-energized, and the dielectric elastomer layer resists the origami material to realize outward bending when energized. The combination of origami materials and dielectric elastomers provides more innovative ideas for programmable and modular design; the actuation behavior of the structure can also be controlled by adjusting the origami material’s creases. In the future, more complex 3D-shaped robots can be designed based on this actuation structure.

Surprisingly, the new type of composite dielectric elastomer material has strong output characteristics. Feng et al. proposed a dielectric elastomer material with large strain and ultra-high energy density (Figure 3C) [69]. Through molecular structure design, this study successfully synthesized a polar fluorinated polyacrylate copolymer (PFED10), which exhibits excellent actuation performance under low-voltage conditions—addressing the core issue that traditional dielectric elastomers require high-voltage driving. Experimental results show the following: The experiment took dielectric elastomer materials of 14 μm and 26 µm as the research objects. When compared with the commonly used VHB4910 material under the same voltage driving, PFED10 achieves a deformation up to 253% of its original size, while VHB4910 only reaches 18%. Even under a low voltage of 550 V, PFED10 still exhibits a deformation of 141% of its original size. By simulating the running posture of cheetahs, micro-crawling robots were designed using both this new composite material and VHB4910. Experimental results show that the robot based on PFED10 achieves better motion performance, including a larger movement range and faster response. Additionally, the planar DEA fabricated by superimposing this material can overcome the pulling force of 1.5 kg of liquid, leveraging its surface expansion deformation. The emergence of this new composite material provides more possibilities for soft robot design.

Duduta et al. [82] designed a soft composite (strain-hardened elastomers + ultra-thin CNT electrodes) with multi-layer stacking, enabling fully flexible DEAs to achieve linear contraction/expansion. This resolves the “flexibility vs. high performance” contradiction of traditional DEAs, yielding fully flexible electric-driven artificial muscles with energy density close to natural muscles. Additionally, Duduta et al. proposed an electrically latched compliant jumping mechanism [83]: a DEA capacitor charging/discharging replaces mechanical locks (reducing moving parts). A dual-material beam (DE and rigid layer) with an initial bent shape enables jumping from both upper/lower orientations. Advantages include suitability for unstructured environments (repeated jumping without precise landing), a simple structure (no pumps/combustion chambers, easy electronic integration), and multi-modal motion (rolling after landing).

Planar DEAs have found application in the biomedical field, with Lee et al. developing a small, controllable dielectric elastomer-driven tip robot for intestinal use [84]—this robot addresses the active navigation challenge of flexible insertion tubes in narrow and tortuous paths, such as the human digestive tract and vascular endoscopes. It is notable that biological intestinal soft robots should be guided by key biological robot literature [85,86,87], with system design centered on the core principles of “safety, functionality, and adaptability”; biocompatibility serves as the entry threshold, requiring the selection of low-toxicity, degradable, or corrosion-resistant base materials along with anti-adhesion and antibacterial surface modifications. Unlike in vitro robots, in vivo robots also need to achieve miniaturized sensing, which can be realized using MEMS components [88], flexible electrodes, and anti-corrosion packaging. Experimental results indicate the robot features miniaturization (with a diameter of several mm), high bendability, biocompatibility, and adaptability to complex liquid environments, operating via a multi-path active navigation mode: when advancing along a flexible pipeline and encountering a path selection, different voltages are applied according to the required path to complete angle bending and path selection. During the research, the team explored three aspects of the robot: for actuation part size optimization, three sizes (15 mm, 22.5 mm, and 30 mm) were tested, with 30 mm leading to reduced stiffness, deviated paths, and smaller bending angles; 15 mm resulting in a large bending angle but limited navigation range; and 22.5 mm identified as the optimal length. For the number of dielectric elastomer layers, experiments on 2-layer, 4-layer, 10-layer, and 20-layer structures showed 20-layer structures having only 1/3–1/4 the bending angle of 4-layer structures, 2-layer structures having a large bending angle but poor stiffness and easy deformation, and 10-layer structures balancing bending angle and shape stability to suit fluid environments. For performance in different fluid environments, a 10-layer tip robot tested in air, olive oil (simulating a blood environment), and 600 rpm stirred water (simulating a turbulent liquid environment) exhibited nearly identical performance in olive oil and air (with sufficient driving force for the simulated blood environment) and better performance than 4-layer robots in rapid currents. In short, the relatively simple structure of planar DEAs allows them to be used as part of the robot body in design, enabling robots to be miniaturized, lightweight, and have relatively simple control methods, but their simple deformation model usually only generates planar motion, making it difficult to replicate the flexible multi-degree-of-freedom deformation characteristics of organisms; future efforts need to optimize the driving performance of planar DEAs and achieve complex motion forms by combining multiple driving units to enhance robot motion performance.

## 4. Saddle-Shaped DEAs

Unlike planar DEAs, saddle-shaped DEAs attach pre-stretched dielectric elastomer films to saddle-shaped substrates (e.g., PET, PVC), with two non-contacting U-shaped reinforcing ribs adhered to the film’s opposite side; the actuator adopts a saddle-like form when the dielectric elastomer film is in its initial contracted state, giving it the name “saddle-shaped DEA”.

### 4.1. Working Principle of Saddle-Shaped DEAs

**Figure 4:**
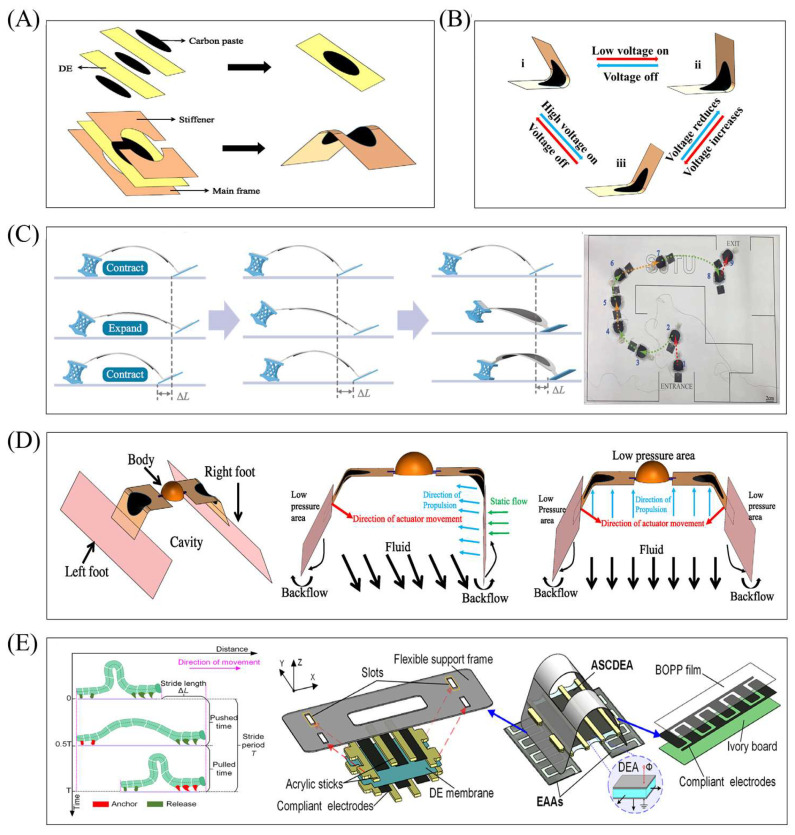
Saddle-shaped DEAs and their robots. (**A**) Structure of saddle-shaped DEA. Reproduced from Reference [56] with permission from *Ocean Engineering*. (**B**) Working principle of saddle-shaped DEA. Reproduced from Reference [56] with permission from *Ocean Engineering*. (**C**) A multimodal motion of a dielectric elastomer robot with reconfigurable hand character sub-feet, including backward, forward, and circular motion, as well as S-shaped trajectory motion. Reproduced from Reference [89] with permission from *Nature Communications*. (**D**) Structure and working method of the bionic crab soft robot. Reproduced from Reference [56] with permission from *Ocean Engineering*. (**E**) The structure of a dielectric elastomer bionic robot simulating the inchworm. Reproduced from Reference [61] with permission from *Advanced Intelligent Systems*.

Saddle-shaped DEAs are another common DEA type, capable of generating a larger output force than planar actuators (Figure 4A). Their key structural differences from planar actuators include tough materials (e.g., PET) attached to both sides of the dielectric elastomer as rigid layers, with the dielectric elastomer (serving as the actuation layer) requiring pre-stretching before assembly, and oval flexible electrodes attached to both sides of the dielectric elastomer, followed by a rigid main frame (with a middle cutout matching the electrode shape) on one side and two non-connected reinforcing ribs (similar in shape to the main frame) on the other. Pre-stretching endows the dielectric elastomer with inward-contracting internal stress, and the combination of the rigid main frame and non-contacting reinforcing ribs gives the assembly a saddle-like initial shape.

The actuator’s operation (Figure 4B) begins with an initial state (Figure 4(Bi)) where pre-stretched dielectric elastomer internal stress results in a small included angle. Applying low voltage expands the dielectric elastomer’s surface area, unfolding the saddle and increasing the included angle (Figure 4(Bii)); further voltage increase maximizes surface area expansion and the included angle (Figure 4(Biii)). Turning off the voltage resets the actuator to its initial saddle shape, and repeating this cycle enables reciprocating opening-closing movements, which have been used to design various robots.

### 4.2. Robots Based on Saddle-Shaped DEAs

This section covers typical saddle-shaped DEA-based robots, highlighting their achievements in multi-modal terrestrial movement, amphibious adaptation, and bionic motion simulation and verifying the actuator’s value in medium- to high-load, cross-environment scenarios. For instance, Wang et al. proposed a dielectric elastomer robot with reconfigurable “hand”-shaped sub-feet (Figure 4C) [89], which combines saddle-shaped DEAs (simulating artificial muscles) with asymmetric feet to achieve multi-modal motion via single-voltage input frequency modulation. Its innovation lies in using the “hand”-shaped feet’s dynamic resonance and chiral torsional effect—adjusting voltage frequency alone enables forward, backward, and circular movements without complex actuation sequences—and integrating shape memory polymer (Vero) thermal response allows S-shaped trajectories and passage through narrow channels at lower heights; experimental results show maximum backward speed of 124 mm/s, forward speed of 112 mm/s, circular motion angular velocity of 0.37 rad/s, and stable S-shaped movement.

Underwater bionic soft robots are another key application: Li et al. developed an amphibious bionic robot mimicking mantis shrimp (Figure 4D) [90], using saddle-shaped DEAs as the main actuation part, asymmetric front–rear foot pads, and innovatively designed cavity-equipped swimming limbs above the feet for multi-modal movement on land, water surfaces, and underwater [91]. For land walking, the saddle-shaped DEA cycles from initial small included angle to large included angle and back when energized—hind foot pads contact the ground for high friction in the small angle state, front feet (lower friction) move forward until front pads contact the ground (high friction) as the angle increases (hind pads lift, hind feet low friction), and hind feet move forward to complete a step when power is off; at 7 kV and 6 Hz, it reaches 170 mm/s and carries 4.6× its own weight. For water surface/underwater movement, repeated DEA opening–closing drives the cavity and swimming limbs (simulating duck webbed feet’s water-pushing effect), achieving 14.4 mm/s at 6 kV and 3 Hz; future upgrades may add water quality sensors and adjust foot pad friction to lower actuator frequency for better climbing.

Li et al. also proposed an underwater bionic crab soft robot (Figure 4D) [56], using two symmetric saddle-shaped DEAs as main actuation parts and two flat PET feet for water drainage and thrust, enabling four multi-modal underwater movements: lateral crawling (independent DEA control replicates crab leg bending, sequential actuation achieves 9.64 mm/s at 5 kV and 1.5 Hz), forward movement (high-frequency 4–7 Hz DEA energization vibrates PET feet for wave-like thrust, 9.55 mm/s at 5 Hz), ascending movement (low 0.2–1.4 Hz voltage enables large DEA bending angle, large PET feet push water for upward thrust, 37.5 mm/s at 4.5 kV and 0.6 Hz), and obstacle avoidance (combines ascending to surface, gravity descent, and one-side actuator energization for lateral offset). Future motion control algorithm optimization will enhance complex environment adaptability.

The saddle-shaped DEA can also simulate the movement of small reptiles: Tete Hu developed a bionic inchworm robot with a saddle-shaped DEA (Figure 4E) [61], whose main body consists of DEA, a flexible support frame, and electro-adhesive feet for multimodal movement. The three-dimensional stable structure composed of the dielectric elastomer and the flexible support frame of this robot is also called the Dielectric Elastomer Minimum Energy Structure (DEMES), which is in the equilibrium state with the lowest total energy of the system when there is no external excitation. In this robot, the DEA simulates the muscle deformation of the inchworm as the main driving force, while the electro-adhesive feet (imitating the hair of reptiles) fix the body on a flat surface (an adsorption force supports wall climbing). The experimental results show that the robot can achieve inverted climbing (0.53 BL/s), vertical climbing (0.71 BL/s), horizontal movement (1.08 BL/s), turning (16.19°/s), and a horizontal carrying capacity of 155 g, demonstrating more complex multimodal motion potential.

Electrically viscous feet (made using electrostatic adsorption technology [92]) are common bionic feet in soft robots. Zhong et al. proposed a bionic crawling robot combining saddle-shaped DEAs with electrically viscous feet [93]. Inspired by inchworm movement (fixation–stretching–fixation–contraction), the robot consists of saddle-shaped DEAs, a 3D scissor structure, and electrostatic adsorption technology—enabling multi-modal adaptive movement. Its crawling has two stages: stretching (rear electro-adhesive feet fixed, DEA deformation unfolds the scissor structure to push front feet forward) and contraction (scissor fully stretched, front feet fixed, rear feet released, actuator reset contracts the scissor to pull rear feet forward). Experiments show paper-based feet move faster on rough paper, while adaptive feet have better adsorption/adaptability; dropping the robot from 15 cm and 40 cm shows normal operation post-landing regardless of initial posture (reducing initial state requirements), and its modular design enables future dual/multi-robot parallel structures and speed difference-based steering for complex environments.

Beyond bionic feet, combining multiple soft actuators improves performance: Yang et al. proposed a small-sized soft terrestrial robot driven by both saddle-shaped DEAs and shape memory alloy (SMA) springs [94], achieving multi-modal motion (rapid running, jumping, combined modes) with a maximum running speed of 2.02 BL/s and jumping height of 1.78 BL, plus excellent slope climbing and load performance; integrating DEAs (high-frequency response) and SMA springs (high energy storage) represents an innovative design.

Combining mechanical structures with saddle-shaped DEAs is another key direction: Wang et al. proposed a flexible breaststroke-inspired swimming robot [95], with an ABS 3D-printed head and actuation part of two saddle-shaped DEAs plus two “adaptive feet” for underwater water-pushing [96]. The DEAs’ upper ends attach to each other, and lower free ends connect to adaptive feet; reciprocating DEA opening–closing enables water-clamping, and adaptive feet mimic breaststroke—unfolding to increase water-facing area/thrust when pushing water, bending under water pressure to reduce area/resistance when recovering—creating a pressure difference that boosts efficiency. Experiments (4.8 kV square wave, 1:2 duty cycle, 0.5–2.5 Hz) show the robot with adaptive feet reaches 73.3 mm/s at 2 Hz (3.15× faster than the one without, 23.3 mm/s) and 76.7 mm/s at 0.5 Hz. These studies break saddle-shaped DEAs’ unidirectional motion limits via special execution structures, cross-material collaboration, and environmental adaptability optimization, verifying their core value in medium- to high-load, cross-scenario soft robots and providing feasible solutions for complex environment robot development.

## 5. Cylindrical DEAs

Compared to planar and saddle-shaped DEAs—simple sheet-like structures with relatively small output forces—cylindrical DEAs are typically formed by winding multiple sheet-like layers, delivering greater overall driving force and deformation. Additionally, the rational configuration of driving electrode positions allows them to generate complex deformation patterns, such as axial elongation and bidirectional bending.

### 5.1. Working Principle of Cylindrical DEAs

**Figure 5:**
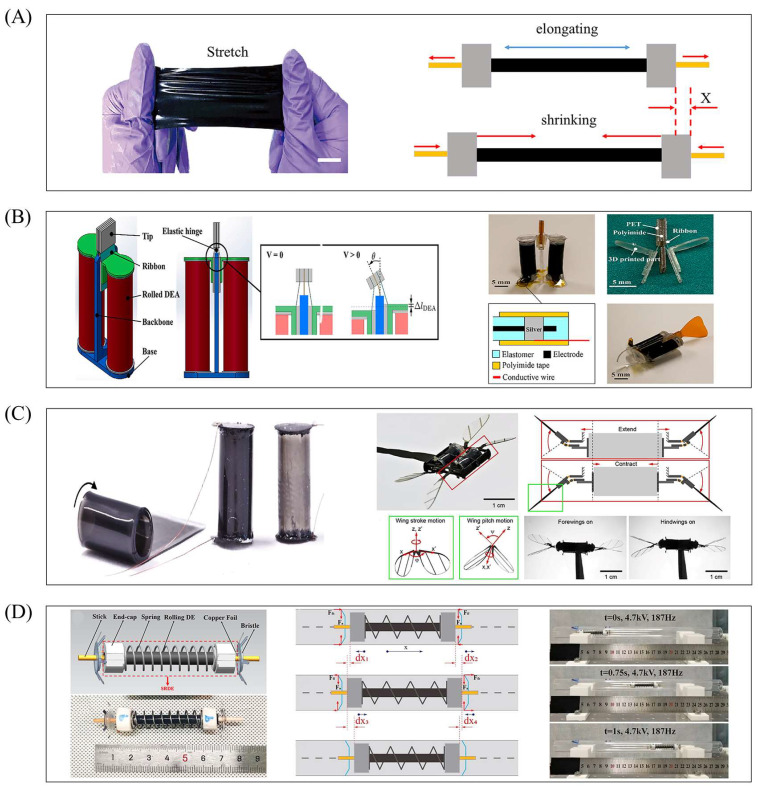
Axially elongating–contracting cylindrical DEAs. (**A**) Structure of the actuator. Reproduced from Reference [102] with permission from *Advanced Science*. (**B**) The structure of the antagonistic actuator of the bionic underwater robot and its tail swing. Reproduced from Reference [97] with permission from *Polymers*. (**C**) The drive structure and working principle of the bionic dragonfly robot. Reproduced from References [98,103] with permission from *Micromachines* and *Advanced Materials*. (**D**) The movement principle of the bristle robot and its pipe crawling. Reproduced from Reference [100] with permission from *Micromachines*.

**Figure 6:**
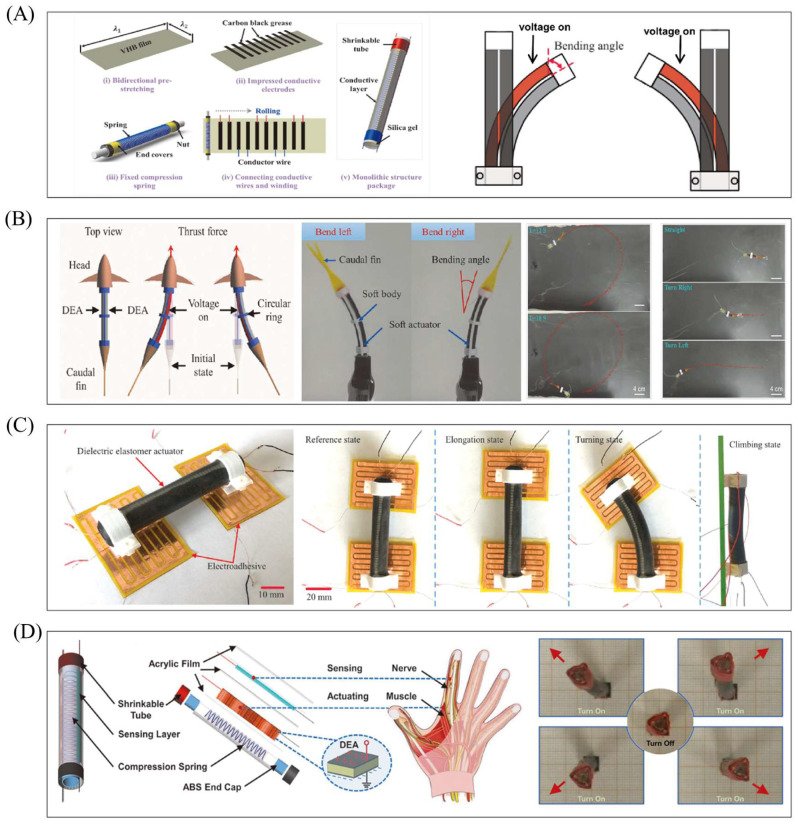
Bending–oscillating cylindrical DEAs. (**A**) Preparation method and structure of the actuator. Reproduced from References [57,60] with permission from *Soft Robotics* and *Extreme Mechanics Letters*. (**B**) The motion principle and multimodal motion of the BCF mode bionic fish robot. Reproduced from Reference [57] with permission from *Soft Robotics*. (**C**) Multimodal movement of bionic caterpillar robots. Reproduced from Reference [60] with permission from *Extreme Mechanics Letters*. (**D**) Cylindrical DEA with sensing and multi-directional movement functions. Reproduced from Reference [107] with permission from *Soft Robotics*.

Figure 5A depicts a cylindrical DEA capable of axial output force, composed of a spring and dielectric elastomer material. Before assembly, the dielectric elastomer is fully pre-stretched and wrapped layer by layer around the spring to form a cylindrical shape, with flexible electrodes (usually flexible carbon grease) attached to the outer surface of each elastomer film layer. Applying voltage causes the layered cylindrical actuator to elongate axially, while powering off resets it to the initial state; repeating the energization–de-energization cycle achieves elongation–contraction movement and generates axial push–pull force. Figure 6A shows another cylindrical DEA for bending and oscillation: its flexible electrodes are attached to the left and right sides as independent, non-contacting units. Energizing one side excites only the corresponding dielectric elastomer (deforming it, while the other side remains dimensionally unchanged), and sufficient deformation bends the actuator toward the unchanged side; powering off reverts it to the initial cylindrical shape. Sequential energization and de-energization of the two sides produce left–right alternating oscillating movements, offering more design possibilities for dielectric elastomer-based soft robots.

### 5.2. Robots Based on Axially Elongating Cylindrical DEAs

As noted in Section 5.1, cylindrical DEAs have two distinct motion forms, with axial elongation (enabling stable linear power output) being ideal for scenarios like underwater propulsion, micro-flight, and pipe crawling. This section focuses on axially scalable cylindrical DEA-based robots, detailing their structural innovations, working mechanisms, and experimental performance across applications (Figure 5B–D). Nagai et al. proposed a rolled dielectric elastomer antagonistic actuator for bionic underwater robots (Figure 5B) [97], combining axially elongating–contracting cylindrical DEAs with an elastic hinge to convert axial tension into bending oscillation; a fish caudal fin attached to the hinge simulates underwater fish oscillation. The two cylindrical DEAs are symmetrically fixed at the bottom, with the elastic hinge midway between their tops—energizing one DEA causes axial elongation and hinge-driven bending, and sequential energization of both achieves continuous oscillation. Experiments used a five-layer dielectric elastomer-wrapped cylindrical DEA, testing the underwater robot (with caudal fin) under 1 kV and 10 Hz, yielding a swimming speed of 0.9 mm/s. Though low driving voltage limits speed, the DEA-mechanical structure combination offers new soft robot design insights; future optimization may improve DEA voltage resistance and modify the hinge/caudal fin for better propulsion efficiency.

Flying soft robots are rare among dielectric elastomer robots, but Chen et al. leveraged cylindrical DEAs’ large driving force and fast response to develop an insect-scale soft dragonfly robot (Figure 5C) [98], combining push–pull flexible hinges with cylindrical DEAs to drive four wings and simulate dragonfly flight [99]. The study explored how the forewing–hindwing phase, driving frequency, and voltage amplitude affect lift—providing a bionic solution for micro air vehicle aerodynamic optimization. In the robot, actuator ends integrate push–pull flexible hinges, with wings under tension in the initial state; voltage-induced axial expansion–contraction of the actuator drives wings to oscillate up and down via the hinges, with two actuators powering four wings (one wing per actuator end). Thrust during ascending flight was used to evaluate performance: in-phase forewing–hindwing flapping achieved a lift-to-weight ratio of 1.32, while tests across 320–410 Hz and 1.45–1.6 kV showed lift peaked at 370 Hz and increased monotonically with voltage—reaching 4.65 mN at 1.6 kV (lift-to-weight ratio 1.49, meeting lift-off requirements). This study provides an experimental basis for insect-scale air vehicle aerodynamic optimization; future work may optimize hindwing design, test inclined flapping surfaces, establish dynamic models, and develop feedback controllers for “take-off (in-phase)–hovering (anti-phase)”-mode switching and autonomous hovering.

Cylindrical DEAs’ shape makes them suitable for bionic crawler robots. Du et al. proposed a soft robot driven by a dual-bristle spring-rolled dielectric elastomer (Figure 5D) [100], capable of multi-scenario movement in pipelines of varying inner diameters, curved pipelines, and on the ground. Its core structure includes an axially elongating–contracting cylindrical DEA and anisotropic bristles at both ends [101]. The special bristle design creates different frictional forces for forward/backward movement (low friction in the bristle orientation direction, high friction opposite). Working principles differ by pipeline–bristle diameter relationship: when the pipeline inner diameter is smaller, bristles are squeezed and bent, with anisotropic friction enabling directional movement—periodic actuator expansion–contraction creates greater rear-end friction, generating a displacement difference between front and rear bristles to drive forward movement after one cycle; when the pipeline inner diameter is larger, unconstrained bristles experience a small bending moment during actuator contraction, and front bristle tips move up while rear tips move down (reducing front friction, increasing rear friction) during elongation—displacement difference pushes the robot forward. Experimental results show maximum speeds of ~1.88 body lengths per second (BL/s) in smaller-diameter pipelines (4.7 kV, 187 Hz) and 2.78 BL/s in larger-diameter pipelines (4 kV, 190 Hz), with long working life and no obvious performance degradation over extended operation. However, the pipeline inner diameter significantly impacts peristaltic speed, requiring further adaptability improvements. These designs fully leverage axially elongating cylindrical DEAs’ linear power output advantages, laying a foundation for applications in complex environments like high-load and long-range scenarios—though small linear strain remains a common issue, calling for future structural optimization or combination with bistable mechanisms to amplify deformation.

### 5.3. Robots Based on Bending–Oscillating Cylindrical DEAs

Bending oscillation—another key motion form—closely mimics the swinging of biological limbs, offering natural advantages in simulating biological propulsion patterns (e.g., fish tail fin swinging, manta ray pectoral fin flapping) and suiting robots requiring multi-modal movements like turning and obstacle avoidance. This section focuses on bending–oscillating cylindrical DEA-based robots, analyzing how they achieve efficient bionic motion and functional expansion via structural design and control strategies (Figure 6A–D). Wang et al. proposed a fast-swimming soft robotic fish driven by bionic muscles (Figure 6A) [57], simulating real fish’s body/caudal fin (BCF) swimming mode with bending–oscillating cylindrical DEAs as bionic muscles—energized DEAs generate oscillation to drive the caudal fin for water propulsion. Tests showed the caudal fin reached a maximum one-way bending angle of 40° at 7 kV (meeting swimming needs) and maximum bending thrust of 14 mN at 7 kV (significantly higher than traditional planar DEAs). Experiments (7 kV, 6 Hz) yielded a maximum swimming speed of 67 mm/s (fast among fish-shaped soft robots), and independent control of bilateral driving units enabled programmable multi-modal motion via a control module: continuous turning (radius ~1.25 body lengths) by maintaining higher voltage on one side, and real-time adjustment of bilateral excitation amplitude for left–right steering switching to complete S-shaped paths. Future optimization may improve body structure for higher efficiency and cable-free swimming. The same team also proposed a bionic manta ray robot driven by bilateral bionic muscles (Figure 6B) [104]; unlike most underwater flapping-wing robots (single-sided oscillation with low efficiency), this design simulates manta rays’ median and paired fin (MPF) propulsion mode [105,106], with bilateral actuators enabling bidirectional oscillation. Experiments used a five-layer cylindrical DEA, which reached a maximum bending angle of 48° at 7 kV; comparative tests showed two-way flapping robots achieved a 54% higher maximum underwater oscillation angle (37° vs. 24° for one-way) at 7 kV, 1 Hz, and 70% higher maximum swimming speed (42 mm/s vs. 25 mm/s), confirming bidirectional oscillation’s ability to enhance underwater performance.

Sensing functionality is critical for adaptive control, and Wang et al. further developed a cylindrical DEA with multi-degree-of-freedom (multi-DOF) movement and integrated sensing [107]. A separate sensing layer (with flexible electrodes) is placed outside the actuation layer to avoid interference, monitoring deformation state via resistance changes for high-precision sensing of bending direction, angle, and strain; multi-electrode distribution enables multi-directional movement, opening new research directions for DEA applications.

Guo et al. proposed a bionic multi-modal soft robot mimicking caterpillars (Figure 6C) [60], driven by a single DEA and two flexible electro-adhesive feet to realize crawling, turning, and climbing [108,109]. The actuator bends toward the non-excited side when one side is energized and achieves axial elongation–contraction when both sides are energized simultaneously—reaching 16.5 mm maximum axial displacement and 108° two-way bending angle at 6.5 kV. Planar crawling works by energizing rear electro-adhesive feet for fixation, applying voltage to both actuator sides for axial elongation, energizing front feet for fixation while de-energizing rear feet and the actuator, and letting the actuator contract to pull rear feet forward (repeating for movement)—reaching 2.38 mm/s at 3.5 kV, 0.8 Hz. Two-way turning involves energizing one actuator side (for bending) after rear foot fixation, then fixing front feet and de-energizing rear feet/actuator to complete turning (maximum speed 1.1°/s), with adjustable voltage excitation time for desired angles and independent bilateral control for different bending directions. Climbing (based on planar crawling) requires raising the electro-adhesive foot excitation voltage to 2.5 kV (overcoming gravity and preventing sliding), with a maximum speed of 2.3 mm/s. Future upgrades may add three-DOF actuation for complex curved surfaces and cable-free design to eliminate wire constraints and expand load capacity.

Bending–oscillating cylindrical DEAs have two core functional advantages: first, their curved oscillation output naturally simulates biological limb swinging (e.g., fish tail fins, manta ray pectoral fins), suiting underwater propulsion and terrestrial crawling; second, they enable multi-functional integration—structural design of separate actuation and sensing layers integrates driving and sensing (supporting robot motion closed-loop control), while multi-electrode distribution expands multi-DOF motion capabilities to enrich robot motion dimensions.

## 6. Conical DEAs

Compared with the previous three DEA types, conical DEAs rely more on structural design for force conversion. They typically consist of a circular rigid frame, a central rigid or flexible top rod, and a DE film—with the film’s edge fixed to the frame and its center firmly connected to the top rod (the rod’s axis is perpendicular to the film plane).

### 6.1. Working Principle of Conical DEAs

**Figure 7:**
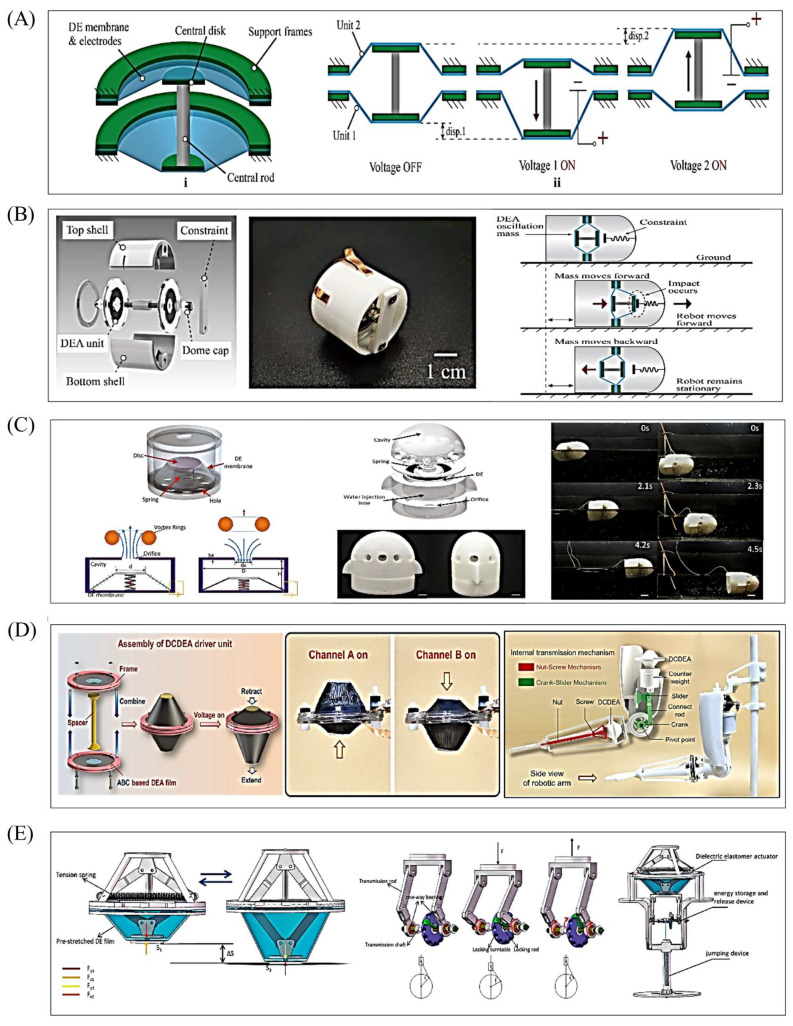
Conical DEAs. (**A**) Structure and working principle of the actuator. Reproduced from Reference [58] with permission from *Micromachines*. (**B**) The structure and motion principle of vibrating crawling robots. Reproduced from Reference [58] with permission from *Micromachines*. (**C**) The structure and motion principle of jet propulsion medium elastomer robots. Reproduced from Reference [62] with permission from *Advanced Engineering Materials*. (**D**) Flexible humanoid robotic arm. Reproduced from Reference [114] with permission from *Advanced Functional Materials*. (**E**) The motion principle and transmission structure of the jumping robot. Reproduced from Reference [115] with permission from *Applied Sciences*.

In its initial state, the actuator is conical. Applying voltage deforms the DE film, but the film’s fixed edge restricts in-plane movement—only allowing the central top rod to lift upward, converting the film’s in-plane expansion into the rod’s axial upward displacement (which increases the conical surface height). Stopping voltage application triggers the DE film’s elastic restoring force, pulling the top rod down to reset to the initial conical shape. Figure 7(Ai) shows a fully symmetrical conical DEA, whose working principle is illustrated in Figure 7(Aii): two parallel DE film layers are connected by a nylon top rod, and their identical size and pre-stretch ratio give the actuator a symmetrical initial structure. Energizing Unit 1 expands its surface area, driving the nylon connector downward; energizing Unit 2 moves the connector upward. Sequential, cyclic energization of the two units enables the nylon connector to reciprocate up and down, realizing the conversion from planar deformation to spatial linear motion.

### 6.2. Robots Based on Conical DEAs

The conical DEA’s force conversion design allows it to adapt to diverse movement needs (crawling, swimming, limb operations). This section focuses on typical conical DEA-based robot systems, detailing their structural innovations, driving mechanisms, and experimental performance to demonstrate the actuator’s value in functional integration and environmental adaptation (Figure 7B–E). Wu et al. proposed a dielectric elastomer-driven impact-type crawling robot (Figure 7B) [58]—a first for soft actuator vibration-impact crawling (unlike common designs inspired by inchworms or caterpillars). A steel cap is attached to one side of the dielectric elastomer, and a constraint is installed at the robot’s head; as the nylon connector reciprocates, it drives the steel cap to collide with the constraint—the impact force overcomes friction to move the robot forward, while no impact force is generated at the tail to ensure one-way motion. Experiments explored constraints’ influence on movement: without constraints, higher voltage increases actuator resonance amplitude but lowers resonance frequency and reduces deviation; with constraints, resonance frequency rises (smaller gaps between the steel cap and constraint cause more obvious frequency deviation); the “impact frequency band” for forward movement is wider than that for backward movement, and the band narrows as the gap increases. At 3.3 kV, 80 Hz, and a 0.6 mm gap, the robot reaches a maximum speed of 12.6 mm/s. Load capacity tests show it handles 0–9.5 g (9.5 g = robot’s self-weight), with speed decreasing linearly—even at 9.5 g, it still moves at 1.8 mm/s. Future research includes developing fully soft robots, testing in viscous environments, and optimizing performance, with potential applications in industrial pipeline inspection, medical capsule endoscopes, and disaster rescue.

Dielectric elastomers also have good water resistance [110]; combining them with a cavity forms a closed space, and voltage-induced film expansion creates pressure differences for water intake and drainage when the cavity is underwater. Inspired by the jet propulsion of cephalopods (e.g., squids, octopuses) [111,112], Tang et al. developed a bionic swimming robot using a dielectric elastomer synthetic jet actuator (Figure 7C) [62]. Addressing limitations of traditional vibrating membrane actuators (e.g., speakers, piezoelectric plates—limited vibration displacement, difficulty simplifying synthetic jet systems), this study uses dielectric elastomers as core materials to optimize synthetic jet actuators, creating a robot capable of moving on water surfaces and underwater. Mimicking cephalopods (which expand their mantle to inhale water and contract to eject it through a funnel), the robot works as follows: in the initial state, the elastic force of the spring and DE film is balanced; applying voltage expands the film, moving the disc to a new equilibrium position; and applying AC voltage induces periodic film vibration around the equilibrium position, continuously squeezing and expanding the fluid in the cavity to achieve jet propulsion. Experiments (5 kV, 15 Hz) show the robot’s average speed is 0.66 body lengths per second (BL/s) on water and 0.43 BL/s underwater—solving traditional robots’ limited environmental adaptability and providing a new scheme for amphibious and underwater robot design.

Dielectric elastomers can also integrate with physical structures like crank–slider mechanisms [113]. Wang et al. proposed a flexible humanoid robotic arm driven by conical DEAs (Figure 7D) [114], which mimics the human arm structure, including a palm for manipulating objects, a forearm for torsion, and an upper arm for lifting. Its transmission mechanisms include an upper arm crank–slider mechanism (the conical DEA’s stroke is transmitted to the crank via a connecting rod, driving the forearm to rotate around the elbow joint and lift the palm) and a forearm nut–screw mechanism (the conical DEA pushes the nut to drive the screw to twist, realizing inversion of the forearm and palm). Experimental results show the forearm can rotate 20.5° around the elbow joint (lifting the palm by 96 mm), and the upper arm can achieve a torsion angle of 108°. Comparative tests of transporting 5 g and 10 g loads (vs. traditional rigid motors) confirm the robotic arm has low energy consumption, energy density close to that of natural muscles, and strong load adaptability. With its high load-to-weight ratio, low energy consumption, high energy density, flexibility, and safety, this conical DEA-driven robotic arm is suitable for human–robot collaboration (HRC) scenarios and can be extended to prosthetics, aerospace, precision instrument assembly, and repetitive operations—providing technical support for next-generation flexible robotic arms.

Luo et al. also proposed a jumping robot capable of energy storage by combining conical DEAs with a crank–slider mechanism (Figure 7E) [115]. The robot’s DEA adopts a multi-layer parallel stacked actuation structure, and stacking 20 layers meets experimental requirements with a peak output force of 30 N. Additionally, the combination of the crank–slider mechanism and a one-way bearing enables energy storage—after 25 energy storage cycles, the robot achieves a jumping height of 45 mm. This highly integrated, functional robot structure provides a design idea for soft robot energy storage. These studies have verified the advantages of conical DEAs in force conversion efficiency and structural compactness while also offering design insights for their subsequent application in miniaturized scenarios (e.g., medical capsule robots) and high-load scenarios (e.g., industrial assembly). In the future, optimizing transmission mechanisms and upgrading packaging technology will further expand their application boundaries.

Planar, saddle-shaped, cylindrical, and conical DEAs—all core driving components in the field of soft robotics—take dielectric elastomer materials as their core. Their basic units share the same working mechanism and common advantages of flexibility, low noise, and high compliance, making them suitable for scenarios with high requirements for drive flexibility and environmental compatibility. However, the four DEA types differ significantly in structural design, motion forms, and applicable fields: Planar DEAs have the simplest structure (usually a sandwich of dielectric, rigid, and conductive layers) and generate bending or in-plane oscillation due to rigid layer constraints, with relatively small deformation and output force. Saddle-shaped DEAs form a hyperbolic surface via pre-stretched DE films and rigid frames; when voltage exceeds a critical value, out-of-plane buckling instability occurs to achieve “switch-type” rapid deformation, with moderate output force and anisotropic deformation capability. Cylindrical DEAs have two motion forms (axial stretching and bending oscillation): the former achieves linear force output through a laminated dielectric film structure, while the latter generates one-sided bending deformation via single-sided electrode excitation, featuring larger output force and a compact structure. Conical DEAs consist of a rigid frame, a central top rod, and a dielectric film; they convert the film’s in-plane expansion into the rod’s axial linear displacement, achieving force output through structural transformation with moderate output force. In terms of applications, planar DEAs (high-frequency response, low-load capacity) suit low-load, high-frequency scenarios; saddle-shaped DEAs (medium load capacity, cross-environment adaptability) can combine with electro-adhesive or anisotropic feet for multi-modal movement; cylindrical DEAs (large output force, dual motion modes) suit high-load or complex motion scenarios; and conical DEAs (unique rigid force conversion structure) can achieve effects unavailable with the other three types.

## 7. Design of Underwater and Terrestrial Robots Based on the Four Types of DEAs

DEAs offer advantages like high energy density, fast response, large driving strain, and easy control, making them widely used in soft robots. However, their high driving voltage poses challenges: commercially available high-voltage power modules are hard to integrate into robot bodies (resulting in most robots being tethered), and even custom-integrated modules increase self-load—limiting DEA use in aerial robots. Currently, DEA-driven robots mainly fall into two categories: underwater and terrestrial. This chapter details the driving characteristics of the four DEA types and explains how to balance actuator configuration and target environment compatibility in robot design, providing guidance for multifunctional robot development and practical requirements for future DEA design.

While DEAs have broad prospects in soft robotics, their performance heavily depends on actuator-environment compatibility. Table 2 summarizes the four DEA types’ actuation characteristics, and the robot design should align with specific performance needs. Below is a classification of dielectric elastomer robots into underwater and terrestrial types, along with how to design robots using these DEAs as main actuation components.

### 7.1. Underwater DE Robots

The underwater environment is complex—deep water, in particular, involves low temperatures and high pressure that directly degrade DEA performance [122,123]. Two key challenges must be addressed: water’s much higher dielectric constant than common DE materials weakens the internal electric field, reducing driving force and strain (an often-overlooked physical issue); seawater’s high ion content and conductivity cause charge leakage (hindering voltage maintenance) and electrode corrosion via electrolytic reactions [124]. Thus, traditional rigid metal electrodes should be replaced with stretchable conductive materials (e.g., carbon grease, carbon glue, liquid metal) to ensure encapsulation integrity and compliance. Additionally, water’s higher density demands efficient movement to overcome fluid resistance, placing stricter requirements on DEA driving frequency, actuator type, and structural design. DEAs are well-suited for building continuous, compliant propulsion systems inspired by biological mechanisms (e.g., fish caudal fin oscillation, pectoral fin undulation, cephalopod jet propulsion). Strict DEA encapsulation is also essential to avoid defects (e.g., pinholes, interface peeling) that cause short circuits or actuator damage; high pressure increases risks of DE material breakdown (requiring avoidance of excessive voltage or local electric field concentration) and actuation instability (wrinkling, collapse). Notably, underwater performance cannot be inferred from air tests, as water pressure and dielectric properties cause significant performance degradation.

**Figure 8:**
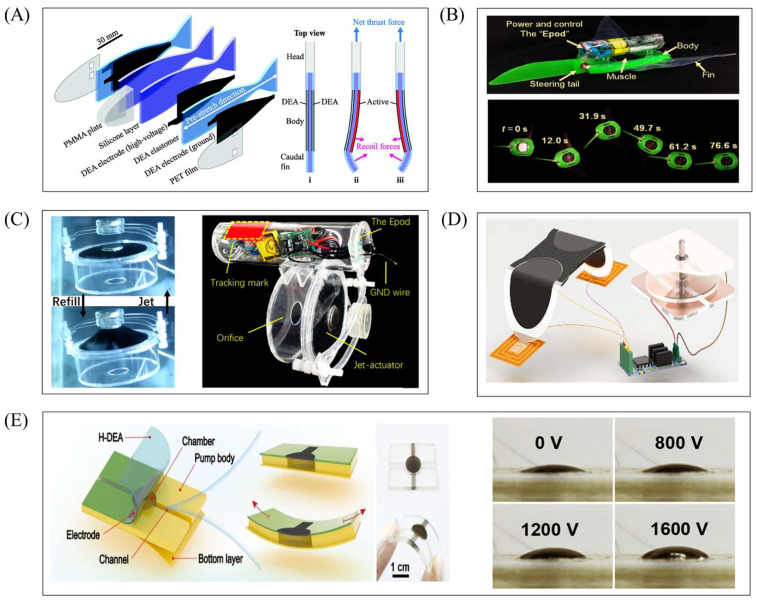
Robots based on different DEAs. (**A**) The structure of a planar DEA-based underwater robot. Reproduced from Reference [125] with permission from *Soft Robotics*. (**B**) The structure of a saddle-shaped DEA-based underwater robot. Reproduced from Reference [126] with permission from *Science Advances*. (**C**) The structure of a conical DEA-based underwater robot. Reproduced from Reference [127] with permission from *Scientific Reports*. (**D**) The structure of a saddle-shaped DEA-based terrestrial robot. Reproduced from Reference [129] with permission from *Advanced Functional Materials*. (**E**) The structure of a planar DEA-based terrestrial robot. Reproduced from Reference [117] with permission from *Advanced Functional Materials*.

Despite these challenges, researchers have achieved efficient underwater swimming via rational robot structure design, control optimization, DEA drive mode matching, and integration with high-efficiency execution structures. For example, Shintake et al. proposed a planar DEA-based soft bionic fish with BCF propulsion (Figure 8A) [125], which utilized four stacked silicone layers to obtain sufficient propulsion force to overcome the influence of the underwater high-pressure environment. A mathematical model based on the Euler–Bernoulli beam (considering non-uniform geometry and hydrodynamic effects) was established to predict the natural frequency in water and the selection of vacuum-guided driving frequencies, thereby reducing the influence of underwater fluid resistance on motion. In addition to the BCF mode, the swimming pattern of fish MPF can also be imitated by relying on DEA. Li et al. developed a manta ray robotic fish driven by a saddle-shaped DEA (Figure 8B) [126]; the saddle-shaped DEA’s actuation mimics manta ray swimming, serving as the core power source to drive pectoral fins for flapping. At 10 kV and 5 Hz, it reaches ~1.45 body lengths per second (BL/s) and integrates a power supply and infrared remote control for cable-free movement (max speed: 0.69 BL/s). As mentioned in the previous text, Wang et al. proposed a bionic manta ray robot based on a cylindrical DEA [104]. Different from this, the cylindrical DEA, with the bionic muscles on both sides of the robot, can be independently controlled. By changing the control sequence, complex multimodal movements can be achieved. During the research on DEAs of different shapes, it was found that the special structure of conical DEAs can change the direction of force transmission. Based on this, Yang et al. designed a conical squid-like soft robot based on a DEA (Figure 8C) [127]. The structural constraints of this DEA are transformed into vertical linear displacements, and the conical DEA can form a closed cavity, completing water intake/spray in the cavity through deformation (imitating the jet propulsion of squids).

From these cases, it can be known that most underwater DEA robots use oscillation or jet propulsion to complete their movements: planar DEA is good at simulating tail fin oscillation (which requires the actuator movement to match the fin/webbed foot movement); Cylindrical DEA (bending/axial stretching) can simulate biological muscles to achieve bidirectional movement well and has great scalability. The conical DEA can be combined with a closed cavity to simulate the shape of jet motion (the deformation of the film changes the pressure of jet propulsion). In addition, the special structure of the conical DEA makes it usually cooperate with actuators such as the tail fin when used as a power source to complete the motion.

### 7.2. Terrestrial DE Robots

Terrestrial DEA robot design requires addressing kilovolt-level voltage risks (electrical breakdown, human safety hazards)—necessitating adequate insulation—and poor air heat dissipation (air has lower heat capacity/conductivity than water [128]). Hysteresis losses during the cyclic DEA driving process can generate heat that is difficult to dissipate, leading to overheating, material aging, shortened service life, etc. Long-term high-frequency full-load driving should be avoided. In addition, how land-based drives can enhance motion efficiency, achieve cable-free motion, and power integration is an important direction for development. The structure must support the actuator and load to prevent the DEA from creeping under static load and optimize performance by combining pre-stretching (to increase the drive strain/energy density and prevent out-of-plane buckling) and rigid/semi-rigid frames (to bear mechanical loads and allow the DEA to focus on driving).

The saddle-shaped DEA combines DEs with a rigid frame to form a saddle-shaped structure, which can amplify the deformation of the elastic body and improve mechanical properties. The movement mode of the saddle-shaped DEA is similar to that of land biological reptiles, making it often used in bionic small crawler robots. Liu et al. proposed a bionic triboelectric soft robot (Figure 8D) [129], using a saddle-shaped DEA as the power source and innovatively integrating a rotating triboelectric nanogenerator (RF-TENG) [130], achieving high-voltage response soft motion driven solely by mechanical energy. The saddle-shaped DEA mimics the movement posture of the inchworm, and the triboelectric adhesive foot (TAF) replicates the inchworm’s “adsorption-fixation-propulsion/traction” mechanism, with a maximum crawling speed of up to 14.9 mm per second.

Generally, the planar DEA motion mode is relatively simple and has a small output force, making it suitable for application scenarios of micro-robots. However, the mechanical properties of the modified composite dielectric elastomer material are relatively strong, which can provide superior mechanical performance for soft robots of the same size as other actuators. Wang et al. proposed a stretchable soft pump driven by a heterogeneous dielectric elastomer (Figure 8E) [117]. This dielectric elastomer material is composed of a multi-layer PHDE-PDMS composite structure, which enhances the service life and mechanical properties (PHDE provides large strain/high energy; PDMS is insulating and resistant to liquid expansion [131]). The maximum flow rate of the all-elastomer pump (20 μm PDMS dielectric layer, weight 1 g, low power consumption) is 3.25 mL/min, and the blocking pressure is 2.75 kPa, which is superior to many commercial micro pumps and large compressors. Moreover, this pump is suitable for various liquids, including high-viscosity fluids such as ethylene glycol.

Cylindrical DEAs can achieve two movement patterns consistent with biological muscle movement by changing the position of electrical stimulation: bending and axial extension. This enables them to realize complex movement forms and ensures that most robots have good force output by using multi-layer stacked DE materials. Li et al. proposed a bionic eyeball structure [118], using three cylindrical DEAs to simulate the extraocular muscles of humans [132]. By adjusting the control sequence and achieving independently controllable DEAs, the limitations of traditional motors/pneumatic actuators/single DEAs were overcome. For the first time, 3D multimodal eye movements (horizontal, vertical, and circular movements) were realized, accurately replicating the movement trajectory of the human eye.

The conical DEA relies on a force conversion structure, so how to combine it with an efficient transmission mechanism is the key to land applications. Youn et al. proposed a flexible micro-gripper driven by helical curved conical DEA [121]. The composite structure of multi-layer DEA superposition and conical helical curved spring overcomes the limitations of traditional conical DEAs, such as easy buckling and difficulty in miniaturization. The spring converts the expansion of the outer surface of DEA into a stable vertical linear drive. At 4.5kV, the maximum displacement reaches 0.36mm, and its maximum output force is 1.2N (0.23N at 0.1Hz), capable of lifting 100.9g of heavy objects. Its performance is superior to that of traditional micro-actuators.

In conclusion, the design of land-based DEA robots first requires the selection of the appropriate DEA type based on the required driving force and DEA movement mode. To enhance movement efficiency, DEA is usually paired with special auxiliary structures (such as electro-adhesive feet, anisotropic hair, unidirectional ratchets, etc.) to improve land-based adaptability and movement stability. In addition, compared with underwater robots, land-driven robots can achieve simple cable-free movement and power integration, which is of great significance for realizing the autonomous movement of soft robots.

## 8. Conclusions and Outlook

This article reviews DEA-based soft robots over the past decade, classifying common DEAs into four categories: planar, saddle-shaped, cylindrical, and conical. It introduces the distinct working principles of these four DEA types, as well as the operating mechanisms, advantages, disadvantages, key innovations, and experimental results of the corresponding DEA-driven robots. Finally, it summarizes and compares the actuation characteristics of the four actuators and explains how to design robots using these DEAs as the main actuation component by classifying dielectric elastomer robots into underwater and terrestrial types.

First, this article analyzes the effects of key factors (voltage, frequency, pre-stretching) on dielectric elastomer performance: voltage directly determines actuation strain and output force but is limited by dielectric breakdown strength; frequency influences the actuator’s dynamic response speed and needs optimization based on the material’s viscoelastic properties; pre-stretching—an indispensable step in DEA fabrication—significantly increases the dielectric breakdown threshold and improves actuation performance.

Building on this, this article classifies DEAs into four basic types, elaborating on their working principles, structural features, and typical applications, respectively: Planar DEAs have a simple structure and are easy to fabricate, making them suitable for small robots requiring high-frequency oscillatory movements (e.g., bionic caudal fin propulsion robots or intestinal navigation robots); saddle-shaped DEAs combine pre-stretching with rigid frames to achieve a large output force and anisotropic deformation and are widely used in crawling robots, jumping robots, and multi-modal motion robots; cylindrical DEAs possess dual capabilities of axial expansion/contraction and bending oscillation, featuring a compact structure and large output force that make them applicable to bionic muscles, aircraft drives, and pipeline robots; and conical DEAs convert in-plane expansion into linear displacement through structural transformation, suiting scenarios requiring linear output (e.g., impact drives, jet propulsion systems, and robotic arms).

Furthermore, from the perspective of two major application environments (underwater and terrestrial), this article systematically summarizes the adaptability and optimization strategies of the four DEA types in robot design: underwater robots require consideration of factors such as high pressure, high dielectric environment, electrode encapsulation, and hydrodynamic effects, while terrestrial robots need attention to issues like heat dissipation, insulation, load support, and motion efficiency.

In general, leveraging their unique advantages of softness, light weight, low noise, and fast response [68,133,134], DEAs are driving the development of soft robots toward more bionic, intelligent, and integrated directions and have demonstrated irreplaceable value, especially in emerging fields such as medical treatment, detection, and collaborative operations.

Despite significant progress in DEA technology for soft robots, it still faces numerous scientific and technological challenges when moving from laboratory research to practical applications: DEAs typically require kilovolt-level high-voltage actuation, which not only poses safety hazards but also increases the size and complexity of power supply systems—limiting their use in portable and embedded systems; dielectric elastomer materials are prone to fatigue, aging, and creep under long-term cyclic loads, while electrode layers may experience detachment, oxidation, or reduced conductivity, seriously affecting the actuator’s service life [135,136]; particularly in air, DEAs generate substantial heat due to hysteresis effects under high-frequency actuation, and untimely heat dissipation leads to performance degradation or even material melting; underwater applications demand extremely high DEA encapsulation standards due to the high-pressure and multi-ion environment, where tiny defects can cause short circuits or breakdown; in terrestrial environments, factors like dust and humidity also affect insulation performance; additionally, DEAs exhibit strong nonlinearity, time-variability, and environmental dependence, making the establishment of an accurate dynamic model and achievement of high-precision control major challenges, and integrating DEAs with sensors, power supplies, and control circuits in limited space to realize true “cable-free” and “autonomous” operation remains a key bottleneck in current research.

To advance the maturity of DEA technology and expand its application scope, future research will focus on several key directions systematically: in terms of materials, efforts will be made to develop new elastomer materials with high dielectric constant and low modulus (e.g., enhancing dielectric properties and reducing driving voltage by incorporating ceramic particles [137], carbon nanotubes [138], or graphene [139]), as well as dielectric elastomer materials with self-healing functions to extend device service life; innovations will also be made in electrodes, including adopting stretchable conductors (e.g., liquid metals, ionic gels, conductive hydrogels) to improve stretchability and environmental stability and advancing multi-functional composite materials to integrate actuation, sensing, and structural support functions; in terms of structural design and manufacturing processes, micro-nano fabrication technologies (e.g., photolithography, 3D printing) will be used to produce micro-DEAs for applications like minimally invasive surgery and microfluidic manipulation, and bionic structural design will draw inspiration from biological structures (e.g., biological muscles, plant tendrils) to enable multi-degree-of-freedom and anisotropic deformation, and controllable, repeatable pre-stretching processes and devices will be developed to enhance device performance consistency; in terms of applications, DEA technology will be further extended to fields including medical robots (e.g., implantable drug delivery systems, soft endoscopes, rehabilitation exoskeletons), detection and rescue robots (soft platforms for extreme environments like deep seas, outer space, and post-disaster ruins), human–robot interaction and collaboration robots (e.g., compliant robotic arms, wearable devices), and bionic/educational robots.

Future work can also focus on improving the performance of soft drives and soft robots: this includes developing diverse drive structures to enable more complex motion forms (e.g., combining energy amplification mechanisms and bistable structures to enhance output force and deformation), optimizing robot body materials to reduce driving voltage and improve output characteristics (e.g., increasing the dielectric constant of DE films and optimizing electrode manufacturing processes), advancing miniaturized high-voltage modules to achieve robot system integration and cable-free movement (facilitating DEA application in aerial robots and even cross-media movement robots), realizing actuator miniaturization (e.g., using 3D printing and MEMS processes for batch fabrication of standard micro-electrodes on DE films to enable microrobot applications, and achieving mass production via automated processes and multi-layer superimposed manufacturing to enhance actuator output force and load capacity for use as the drive core in large-scale and industrial equipment), and developing hybrid drives—by combining functional materials (e.g., integrating electronic control and magnetic control technologies to enable both electric and magnetic drive). A typical hybrid drive design involves doping soft magnetic particles or permanent magnetic particles into the electrode or elastic matrix of dielectric elastomers to construct an electro-magnetic dual-response material composite system, with the doping volume fraction controlled between 5% and 30% (too low a fraction results in weak magnetic response, while too high a fraction causes a sharp increase in the elastomer’s elastic modulus and reduced deformation rate). Ultimately, this design retains the advantages of fast response and large deformation of dielectric elastomer electric drive, while supplementing large thrust and strong load capacity via magnetic drive to achieve the synergistic effect of electric drive speed control and magnetic drive force control.

## Figures and Tables

**Table 1:** Performance comparison of different soft actuators.

	Actuation Mechanism	Response Speed	Maximum Strain	Typical Motion Modes	References
SMA	Martensitic transformation	Second level (1 s–5 s)	60%	Linear motion, bending, twisting	[40,41,42]
Hydrogel	Ion migration and solvent redistribution	Second level (140 ms–2 s)	1200%	Volume expansion and contraction, bending	[43,44,45]
DEAs	Maxwell’s stress effect	Millisecond level (10 ms–500 ms)	1600%	Planar expansion, linear contraction, bending	[46,47,48]
Pneumatic	Pressure change	Millisecond level (100 ms–2 s)	300%	Bending, stretching, and twisting	[49,50,51]
IPMCs	Ion migration induced by an electric field	Second level (500 ms–5 s)	5%	Large-amplitude bending and swinging	[52,53,54]

**Table 2:** Characteristics of the four types of DEAs.

	Maximum Strain	Output Force	Response Frequency	Environmental Adaptability	Manufacturing Difficulty	Typical Motion Modes	Reference
Planar Type	400%	8.8 N	100 Hz	Poor	Easy	Bending, linear motion	[55,116,117]
Saddle-Shaped Type	500%	0.6 N	30 Hz	Medium	Medium	Bending, swinging	[61]
Cylindrical Type	500%	3.9 N	400 Hz	Excellent (easy to encapsulate)	Difficult	Bending, swinging, linear motion	[60,102,118]
Conical Type	400%	30 N	80 Hz	Good	Medium-Difficult	Linear motion	[119,120,121]

## Data Availability

No new data were created or analyzed in this study.
